# Student and Preceptor Perceptions of the Summer Clinical Practicum: An Assessment of the First Year Clinical Skills Capstone

**DOI:** 10.15694/mep.2017.000140

**Published:** 2017-08-02

**Authors:** Leslie Beitsch, Karen Geletko, Gail Bellamy, Andree Aubrey, Karen Myers, Luckey Dunn, Alma Littles, John Fogarty

**Affiliations:** 1 Florida State University College of Medicine

**Keywords:** clinical skills, primary care, doctor patient relationship

## Abstract

This article was migrated. The article was marked as recommended.

**Objective:** As the first new medical school chartered in the 21 ^st^ century, FSUCOM adopted a unique approach to medical education. Beyond the expected basic science courses, first year students participate in a year-long Doctoring and Clinical Skills Course. The first year culminates in a three week Summer Clinical Practicum (SCP) emphasizing mission centric populations. We designed a survey to assess medical student and clinical preceptor impressions of the SCP for purposes of quality improvement.

**Methods:** Students in their second, third, and fourth years and their preceptors were included in a cross sectional descriptive study. All participants responded to an electronic web based survey developed and administered in Summer 2016. The overall student response rate was 32%, while 53% of preceptors participated.

**Results:** Survey results indicated that the SCP first year capstone experience is highly valued by students and preceptors alike. We found a high concordance of perceptions between student cohorts spanning three years and faculty preceptor respondents. For purposes of programmatic planning and quality improvement there was strong support for maintaining the current length for the SCP at three weeks. Additionally, survey findings appear to support ongoing perceived benefit from the early clinical exposure summer experience, with students agreeing that the SCP helped focus their learning as M-2s, and influenced their readiness for subsequent clinical clerkships.

**Conclusion:** Our study supports the growing body of knowledge that early clinical experience for matriculating medical students is not only feasible, but desirable, and has lasting effects throughout their undergraduate medical education.

## Introduction

The conceptual framework supporting early clinical exposures for medical students is not new. In fact, the Council on Medical Education and Hospitals report at the 1921 American Medical Association annual meeting called for revisions of medical school curriculum, including earlier clinical activity. (Eaglen) Florida State University College of Medicine (FSUCOM) took these and similar subsequent recommendations under serious consideration as the initial curriculum was developed. Moreover, embedded in the institutional DNA, early clinical experience has been a cornerstone of the FSUCOM curriculum since its inception in 2000.

As the first new medical school chartered in the 21 ^st^ century, FSUCOM adopted a unique distributive model of medical education, utilizing six regional campuses, two rural campuses (one serving migrant farmworker families), and community faculty in a largely outpatient apprenticeship model. ( Fogarty, Littles, Romrell, Watson, & Hurt, 2012; Hurt & Harris, 2005) Heavy emphasis in the curriculum is also placed upon fulfilling the college’s mission regarding responsiveness to community needs: “..especially through service to elder, rural, minority, and underserved populations.” Additionally, the admissions process seeks to develop a pool of highly qualified applicants who have demonstrated commitment to these same groups, coupled with a curriculum promoting entrance into primary care specialties. ( Hurt & Harris, 2005)

To accomplish successfully the objectives outlined, clinical skills are first introduced early during the initial summer semester of the three semester first year. In an effort to decompress the traditional first year, while also allowing more time for early clinical experience and community service activities, students begin class in late May of the M-1 year. Beyond the expected basic science courses, the students participate in a year-long Doctoring and Clinical Skills Course. This introduction to patient care skills includes didactic and simulated patient sessions focusing on interviewing and physical examination skills, and office preceptor experience with community physicians trained in teaching them the skills, knowledge, and attitudes required for a robust doctor-patient relationship. The first year calendar culminates in a three week Summer Clinical Practicum (SCP) emphasizing mission centric populations (geriatrics, rural health, and urban underserved). To the greatest extent possible, students are assigned to a clinical faculty member associated with one of the regional campuses whose practice includes medically underserved populations or is located in a health professions shortage area. This intensive capstone session presents an experiential learning opportunity reflecting all of the skills acquired during Doctoring. From the outset in 2004-2005, the SCP has been highly rated by students.

The current survey was designed to assess medical student and clinical preceptor impressions of the SCP to determine if they are meeting the program’s learning objectives. The data gathered from the survey reflects students’ and clinical preceptors’ perceived quality and value of the SCP. Students in their second, third, and fourth years were included in an effort to ascertain the impact of the SCP immediately following the experience and to determine if the experience still holds value for students as they continue further in their medical education. Preceptor input was necessary to validate and triangulate student perspectives. The feedback obtained from the survey will be used to guide improvements in the program.

## Methods

During the Summer of 2016 we administered a cross sectional, descriptive study of medical students’ perceptions about their Summer Clinical Practicum (SCP) experience. Responses to a brief self-administered survey were solicited via Likert scale, multiple choice, and open-ended questions focused on the SCP competency domains of patient care, medical knowledge, practice-based learning, communication skills, professionalism, and system-based practice. The survey is available upon request. In an effort to broaden perspective, the study utilized three successive classes of medical students who have participated in and completed the SCP, and the preceptors who served as faculty during the 2016 SCP. Inclusion of three student classes allowed for examination of possible change in perspective about the SCP as students progressed in medical school. One or more questions differed by cohort group, reflecting the time differences when students had the SCP experience or the faculty role as preceptors. Students and preceptors were invited to participate in the study via email from the dean, which included the link to the anonymous Qualtrics survey instrument, allowing all data to be collected electronically without possibility of identifying the respondent. Invitations were sent via email to the entire classes and the individual preceptors on three successive weeks, encouraging their completion of the survey. No enrolled students or preceptors were excluded from the study. The Institutional Review Board at Florida State University approved this study.

Analysis of surveys was implemented using SAS software version 9.4 for Windows, utilizing the SAS procedure FREQ to generate descriptive statistics. All questions were framed in the affirmative, and asked whether the question reflected the respondent’s viewpoint. Likert scale questions were assessed as follows: Strongly Agree=5; Agree=4; Neutral=3; Disagree=2; and Strongly Disagree=1, and results are expressed as means. Independent t tests and Anova assessed response differences of the continuous variables across student and preceptor cohorts, with statistically significant findings expressed at the p≤.05 level. Short responses to open-ended questions were not included in the descriptive statistics.

## Results

The response rates for the study differed somewhat by cohort, with the highest rate among those just completing the SCP (Class of 2019: 40%; Class of 2018: 20%; Class of 2017: 32%) for an overall rate of 32%. Similarly, the preceptors were highly motivated, with a response rate of 53%. Second year students (Class of 2018) were surveyed during the Step 1 testing period, which was also when many students were relocating to regional campuses for third year clerkships. Students and preceptors were asked about their agreement with a number of statements regarding their SCP experience. These perceptions are depicted in Table 1. Most responses were favorable with means falling in the Agree-Strongly Agree range, with substantial concordance across cohorts of students and preceptors alike. Notable exceptions are clustered within the domain of survey questions related to chronic disease management, in which student responses were trending as less favorable. The results as summarized here are generalized. Some survey questions had differences across the responding cohorts, and where these differences are statistically significant, it is indicated with an asterisk in p value column of Table 1.

Generally students and preceptors concurred that the SCP experience allowed them to be exposed to a wide variety of patients and clinical conditions. Moreover, the patients within the SCP practices reflected the FSUCOM mission (racial and ethnic minorities, rural, and elderly populations). Skills development and knowledge acquisition were also perceived as supported through the SCP experience. Students were likely to practice their life-long learning skills following clinical encounters; they applied their basic sciences to clinical care; and practiced documentation of clinical encounters. Preceptors were slightly more optimistic in their perceptions.

Mentorship is a critical component of the SCP model, serving as an early introduction for the FSUCOM transition to the apprenticeship of the clinical clerkships. Students and preceptors were asked about adequacy of preparation for the SCP, opportunities for feedback, professional growth/independence, and one-on-one time for teaching and information exchange. All aspects were viewed positively, with preceptors’ opinions somewhat more favorable. First year students were less likely than others to feel their preceptor was familiar with their current level of clinical skills, possibly reflecting recent curricular reform at FSUCOM.

In the important domain of SCP value, students and preceptors reached consensus that they would recommend a SCP-like experience for all first year medical students. Most felt it was one of the best learning opportunities of the first year, although fourth year students were slightly less enthusiastic on that point. However, when queried about the SCP value in relationship to preparing them for third year clerkships, fourth year students were more supportive (4 of 5 on the 5 point Likert scale). One student noted “..the SCP was instrumental in making me the student I am today.. SCP gave me the foundation and the confidence, as a first year medical student to not only think critically, see patients independently and learn medicine in a real-life setting, but to also know I had the potential to get so much better at the art of medicine.” Rising third and fourth year students were asked if what they experienced during the SCP helped them focus their learning during the second year of medical school. Their consolidated responses fell between neutral and agree. All cohorts agreed that they demonstrated improvement in history and physical examination performance and had more confidence in building the clinical relationship. Students and preceptors expressed a large preference for the current three week SCP rather than reducing it to a two week experience. Individual comments (data not shown) regarding the preference for three versus two weeks were related to growing student confidence and learning as they acquired comfort within the clinical setting and gained greater independence with each intervening week.

Since inception there has been a close nexus between the FSUCOM SCP and the Area Health Education Center Program (AHEC). Recent emphasis of the AHEC program in Florida on chronic disease, specifically tobacco prevention and treatment, has also influenced the SCP curriculum. Survey results suggest this component may be less successful than other aspects of the program, especially for the current first year cohort. Compared with previous years, current first year students did not feel as well prepared to intervene with patients who have tobacco dependency. They also felt less confident in their knowledge about how to successfully refer such patients to other sources of care available statewide. Few students asked their preceptors if they could be involved with patient education regarding chronic disease, although most students felt such opportunities arose over the course of the SCP.

## Discussion

Several findings from the present study merit emphasizing. The SCP first year capstone experience is highly valued by students and preceptors alike. Moreover, there is high concordance of perceptions between student cohorts spanning three years and faculty preceptor respondents. For the vital purposes of programmatic planning and quality improvement there was strong support for maintaining the current length for the SCP at three weeks rather than compressing it into two weeks. There was general agreement across all cohorts and preceptors for this position, even though in the case of the students, it shortened their summer vacations following a three semester first year. Additionally, survey findings appear to indicate continued, longer duration perceived benefit from the early clinical exposure summer experience. Students agreed that the SCP helped focus their learning as M-2s, and influenced their readiness for subsequent clinical clerkships. When interpreted in the context of other responses, these findings follow logically. Students (and to a greater extent the preceptors) felt they were able to apply basic sciences in the clinical setting. It was also consistent with their increased confidence in history taking and the physical examination as well as establishing the doctor-patient relationship.

Littlewood and colleagues conducted a systematic review of medical school programs offering early clinical and community based experiences. Casting a wide net, 38 studies met their criteria, with most from the U.S. (Littlewood et al.) They concluded that students in such programs were more likely to select primary care as a career choice, and were positively disposed toward rural settings. They also noted a potential selection bias among participating students, which might influence the conclusions. Students were more confident in clinical skills and learned more about the social contexts in which medicine is practiced. Neiman et al employed comparison groups among students at the University of Texas, with roughly half electing a four week primary care preceptorship, while half did not. (Nieman et al.) Students participating in the clinical experience performed better on their objective structured clinical examination. Again selection bias may be a factor.

Other schools have utilized programs similar to the SCP in terms of content and timing, but may have somewhat differing underlying purposes. The University of Missouri takes a multi-faceted approach toward increasing the rural physician pipeline, which includes a rural early clinical experience “elective” lasting 4-8 weeks that resembles SCP. ( Kane et al., 2013) Their evaluations reveal a greater likelihood of student selection of primary care residencies among those who opted for the rural summer program. A similar multi-faceted program with rural emphasis shared among several Australian medical schools also demonstrated higher rates of student entry into primary care. ( Daly, Perkins, Kumar, Roberts, & Moore, 2013; Roberts et al., 2012) Increased entry into primary care residencies and practices, although not solely focused on rural, is likewise an objective shared by FSUCOM, but one not examined in this study.

Although other medical schools have developed curricula incorporating early clinical exposure for their students ( Kamien, 1990; Secundy & Lloyd, 1974; Steele et al.), the FSUCOM SCP model shares many similarities with a program developed earlier at the University of Florida (UF). ( Rooks, Watson, & Harris, 2001) Both were originally developed with close ties to Area Health Education Center programs, and emphasize clinical experience in primary care settings. The UF program falls between first and second semesters of the first year, when students have had considerably less clinical skills development. Nonetheless, student satisfaction with the early clinical exposure is high at both institutions. ( Rooks et al., 2001) Participation was active rather than passive, in contradistinction with earlier premedical shadowing roles. Rooks, et al also cited that students had greater appreciation of the relevance of the preclinical curriculum to future medical practice. ( Rooks et al., 2001) This was consistent with our responses from third and fourth year students. The development of a new integrated curriculum in the medical school in the past 2 years may also have influenced the positive responses from the latest class and their preceptors since they had more exposure to clinical concepts along with their first year basic science instruction.

The current study has several limitations, offset in part by strengths in its design. First, as with the majority of the research studies cited herein, there is no control group. However, three consecutive student cohorts were included, as were clinical preceptors for this point in time survey. Second, as with prior studies the FSUCOM survey must be interpreted through the lens of potential selection bias. However, in this case, it is most pronounced during the admissions process itself-with a strong predisposition for admittance of students intending a career in primary care-and not in the survey administration. Moreover, all students participated in the SCP, and response rates were high. Third, social desirability bias may also have played a role in student and preceptor responses. Finally this was a single point in time survey, but nonetheless it allowed a longer term assessment because it included student perspectives immediately following the SCP, one year afterwards, and two years later. The high degree of concordance between student and preceptor perceptions further validates the findings that we report.

In conclusion, our study supports the growing body of knowledge that early clinical experience for matriculating medical students is not only feasible, but desirable. Students indicated that they highly prize the SCP and derived many benefits from participation. Importantly we found, as have others, that the value endures as students enter clinical rotations. The opportunity to be embedded within a primary care practice for three weeks (superior to two weeks, if not optimal), facilitated the achievement of several intermediate learner milestones (linkage and application of their first year basic science education with clinical settings/presentations; improving ability and confidence performing clinical histories and physical examinations; comfort and skill establishing the doctor-patient relationship; and exposing them to FSUCOM mission based patients within a social context).

**Table 1.** Student and Preceptor Perceptions of the Summer Clinical Practicum

Please select how much you agree with each statement regarding the Summer Clinical Practicum experience.	Class of 2019 (n=50)	Class of 2018(n=25)	Class of 2017(n=43)	Preceptors(n=47)	Total n	P value
**Patient Population and Mission Centricity**						
**I/My student saw a wide variety of patients and clinical conditions during the SCP.**	**4.02 (50)**	**4.00 (24)**	**3.91 (43)**	**4.81 (47)**	**164**	0.00035*
**I saw patients in my preceptor’s office/patients seen in my practice that represented our COM mission (specifically, patients from rural areas, elders and/or racial/ethnic minorities).**	**4.36 (50)**	**4.50 (24)**	**4.30 (43)**	**4.55 (47)**	**164**	**0.559278**
**Skills Development and Knowledge Acquisition**						
**I sought additional medical information about patient conditions seen during SCP after the clinical encounter.**	**4.58 (50)**	**4.42 (24)**	**4.37 (43)**	**4.57 (47)**	**164**	**0.371264**
**I/My SCP student was able to apply the basic sciences I/he/she learned in the first year of medical school to patients I/he/she cared for during the SCP.**	**4.10 (50)**	**4.08 (25)**	**3.95 (43)**	**4.57 (47)**	**165**	0.01477*
**I/My student was able to practice clinical encounter documentation skills during SCP**	**4.12 (49)**	**4.13 (24)**	**3.42 (43)**	**4.55 (47)**	**163**	<.001*
**Mentorship**						
**My SCP preceptor/I was familiar with my/my students level of clinical skill.**	**3.78 (50)**	**4.17 (24)**	**4.35 (43)**	**4.49 (47)**	**164**	0.001768*
**My preceptor/I made time in the schedule to solicit thoughts and impressions of what I/my student had seen that day.**	**4.20 (50)**	**4.04 (25)**	**4.23 (43)**	**4.57 (47)**	**165**	**0.100568**
**I was encouraged/I encouraged my student to learn new clinical skills during the SCP.**	**4.28 (50)**	**4.33 (24)**	**4.26 (43)**	**4.72 (47)**	**164**	0.047753*
**I was given/my staff and I gave feedback during SCP by my preceptor or staff.**	**4.14 (50)**	**4.25 (24)**	**4.42 (43)**	**4.55 (47)**	**164**	**0.093352**
**I was given/I gave my student more independence/responsibility each week of the SCP.**	**4.08 (49)**	**3.96 (24)**	**4.21 (43)**	**4.49 (47)**	**163**	**0.12492**
**Overall, I felt that I/my student was well prepared to participate fully in the SCP.**	**4.26 (50)**	**4.21 (24)**	**4.19 (43)**	**4.68 (47)**	**164**	0.016111*
**Value of SCP Experience**						
**I increased my confidence in/My student(s) was able to demonstrate significant improvement performing a history and physical examination during the SCP.**	**4.22 (50)**	**4.33 (24)**	**4.45 (42)**	**4.68 (47)**	**47**	**0.0683**
**The SCP experience increased my/my student(s) confidence in establishing the patient provider relationship.**	**4.36 (50)**	**4.42 (24)**	**4.35 (43)**	**4.70 (47)**	**164**	**0.112765**
**The SCP was one of the best learning opportunities of the first year curriculum.**	**4.16 (50)**	**4.13 (24)**	**3.70 (43)**	**4.45 (47)**	**164**	0.013067*
**Two weeks of the SCP would be sufficient to meet the assigned clinical learning objectives.**	**3.28 (50)**	**3.08 (24)**	**3.35 (43)**	**2.74 (47)**	**164**	**0.060939**
**Three weeks of the SCP were sufficient to meet the assigned clinical learning objectives.**	**4.34 (50)**	**4.58 (24)**	**4.28 (43)**	**4.09 (47)**	**164**	**0.149103**
**What I learned during SCP helped to focus my learning during my second year of medical school.**	**NA**	**3.58 (24)**	**3.26 (43)**	**NA**	**67**	**0.331136**
**Having completed the third year clerkships, I value the SCP learning experience.**	**NA**	**NA**	**4.00 (43)**	**NA**	**43**	**NA**
**I would recommend an experience like the SCP for first year medical students attending any medical school.**	**4.82 (50)**	**4.56 (25)**	**4.56 (43)**	**4.89 (47)**	**165**	0.015042*
**Chronic Disease Management**						
**The tobacco training I received in the Spring prepared me to counsel patients who used tobacco during the SCP.**	**2.60 (50)**	**3.17 (24)**	**3.50 (43)**	**NA**	**117**	0.00069*
**I know/the SCP student knew how to refer patients who are ready to quit tobacco to treatment services (percent “YES”).**	**58% (29)**	**75% (18)**	**74.4% (32)**	**55.3% (26)**	**117**	0.022626*
**During SCP, I was given opportunities/The student(s) was able to demonstrate the ability to counsel patients regarding chronic disease self-management or their tobacco use (percent “YES”).**	**58% (29)**	**66.7% (16)**	**58.1% (25)**	**74.5% (35)**	**117**	**0.462253**
**I asked my preceptor if could counsel patients regarding chronic disease self-management or their tobacco use (percent “YES”).**	**9.5% (2)**	**14.3% (1)**	**33.3% (6)**	**NA**	**46**	**0.319021**
**I would find faculty development (i.e. CME) helpful to my role as a preceptor.**	**NA**	**NA**	**NA**	**3.77 (47)**	**47**	**NA**

## Take Home Messages

• The first year capstone experience is highly valued by students and preceptors alike.
• There was a high concordance of perceptions between students and faculty preceptor respondents.
• Findings support ongoing perceived benefit from the early clinical exposure summer experience, with students agreeing that the SCP helped focus their learning as M-2s, and influenced their readiness for subsequent clinical clerkships.
• Our study supports early clinical experience for matriculating medical students is feasible and desirable.

## Notes On Contributors

All contributors are faculty at Florida State University College of Medicine:

Leslie Beitsch, MD, JD

Karen Geletko, MPH

Gail Bellamy, PhD

Andree Aubrey, MSW, LCSW

Karen Myers, ARNP

Luckey Dunn, MD

Alma Littles, MD

John Fogarty, MD
