# Supplementary material for: Student and Preceptor Perceptions of the Summer Clinical Practicum: An Assessment of the First Year Clinical Skills Capstone
Source: MedEdPublish (2016). 2017 Aug 2;6:140. [Version 1] doi: 10.15694/mep.2017.000140 (PMC10885285; doi:10.15694/mep.2017.000140)
Supplement: Peer review history

# Reviewer report: Reviewer response for version 1

**DOI:** 10.21956/mep.19325.r28214

**Published:** 2019-04-06

**Authors:** Ken Masters

**Affiliations:** 1 Sultan Qaboos University

This review has been migrated. The reviewer awarded 3 stars out of 5 This is a useful paper on the student perception of their experience of a new medical school. Although the results are very localised, they do give some valuable insight into the students’ experience.There are few things that I would like to see addressed in the paper:• Abbreviations (e.g. FSUCOM) should be written out in full the first time they are used, including in the Abstract.• The authors describe their medical school as “the first new medical school chartered in the 21st century.” They would need some sort of data backing up the statement to indicate that, worldwide, FSUCOM was the first new medical school chartered (unless they are using “chartered” in a particular sense).• The authors write “Similarly, the preceptors were highly motivated, with a response rate of 53%.” The “similarly” applies to the student responses. Given that student responses were as low as 20% (and a mean of 32%), that does not imply a high level of motivation.• The low response rate, even if similar to other surveys, needs to be acknowledged as a limitation.• It would be useful if the authors could elaborate on the steps taken to improve future student experience with the chronic disease experience.

Reviewer Expertise:

NA

No decision status is available

# Reviewer report: Reviewer response for version 1

**DOI:** 10.21956/mep.19325.r28213

**Published:** 2017-08-04

**Authors:** Trevor Gibbs

**Affiliations:** 1 AMEE

This review has been migrated. The reviewer awarded 4 stars out of 5 A very interesting and well-written paper, which I think will want the reader to learn more about this exciting course. As the authors state, there is a wealth of literature that supports the benefits of early clinical exposure; this paper clearly adds to that., as well as showing the benefits of a capstone activity, which I feel is not that common amongst programmes.I particularly liked the social responsiveness element- I would encourage the authors to follow up these students and their effect upon healthcare of the community- changing social responsiveness to social accountabilityI would ask the authors one particular question, which I think could be asked of other similar papers. This innovation appears to have been carried out within an organisation in which resources appear freely available. Could this innovation be transplanted to a less well resourced, less well developed country or are we making the world of medical education much less of a level playing field ?I feel that this paper would be usefully read by all those involved in curricula planning and hope that they would be prepared to share their innovative curriculum with others

Reviewer Expertise:

NA

No decision status is available
